# Well-differentiated squamous cell carcinoma of the clitoris: a rare case report

**DOI:** 10.11604/pamj.2024.48.153.44531

**Published:** 2024-08-05

**Authors:** Shivali Kalode, Kishor Hiwale

**Affiliations:** 1 Department of Pathology, Jawaharlal Nehru Medical College, Datta Meghe Institute of Higher Education and Research, Sawangi (Meghe), Wardha, Maharashtra, India

**Keywords:** Clitoris, well-differentiated squamous cell carcinoma, keratin pearls, case report

## Abstract

Squamous cell carcinoma (SCC) can impact many different organs, including the vagina and vulva skin, that are covered with squamous epithelial lining. With a yearly prevalence as low as 2.6 per 100,000 people, Vulvar carcinoma is a rather uncommon type of gynecological neoplasia. It commonly manifests in people 70 years of age and older. Our case study is about the well-differentiated squamous cell carcinoma (WDSCC) of the clitoris in a female patient who is 65 years of age. However, there is an increasing number of women aged 35 to 45 who are being diagnosed with this type of cancer. The biopsy of the clitoris revealed severe dysplastic squamous epithelial cells, diagnostic for WDSCC of the clitoris. In the past, the gold standard for treating even mild invasive vulva carcinomas involved a radical vulvectomy with a wide margin of tumor excision, an en bloc resection of the inguinal and frequently the pelvic lymph nodes. At the moment, a less drastic and more customized course of action is advised.

## Introduction

Clitoris tumors are extremely uncommon but significant because of their aggressive clinical course, which can result in early mortality. Approximately 4% of all gynecologic malignancies are vulvar carcinomas, of which localizing to the clitoris makes it a rarity [1]. Different clinical appearances, such as ulcerated plates or lesions resembling warts, are possible for vulvar SCC [2]. Women usually present with progressive vulvar pruritus and tightening of the skin leading to dyspareunia [3]. Based on predisposing factors, vulvar cancer can be divided into two groups: the first kind is associated with an human papillomavirus (HPV) infection and primarily affects younger people. The second group, more common in older women without neoplastic epithelial diseases, is unrelated to HPV [4]. Every suspected vulvar lesion needs to be biopsied to exclude invasion. After being discovered, squamous cell carcinoma is the most common subtype [5]. Aggressive clinical behavior and complex histology pose significant challenges to accurate identification and successful treatment strategies [6].

Vulvar cancer is still under debate, however broad local excision is the recommended course of action given the absence of evidence of spontaneous spread and strict follow-up [7]. All of the patients, however, passed away within three years of diagnosis, either from their immunosuppressive sepsis or cancers [8]. Accurate long-term monitoring should be started for patients, particularly if they are younger and have small tumors, as recurrence or re-occurrence can happen even years after diagnosis [9]. According to the results, patients with high-grade squamous intraepithelial lesions may require risk stratification to avoid overtreating their lesions. It is also important to promptly identify and classify differentiated vulvar intraepithelial neoplasia precancerous lesions [10].

## Patient and observation

**Figure 1:**
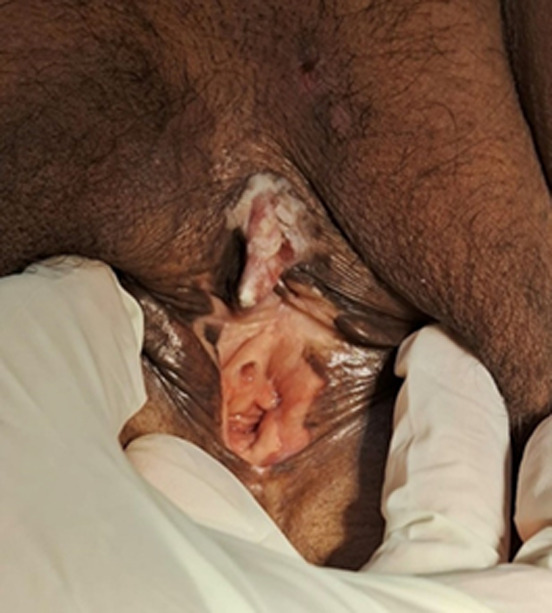
a firm fungating mass over the clitoral area

**Patient information:** we are reporting a case of well-differentiated squamous cell carcinoma of the clitoris in a 65-year-old female who presented in the gynecological outdoors with a complaint of a small swelling in the perineal region for 2 years which progressively increasing in size with time and associated with itching (Figure 1).

**Clinical findings:** on examination, a firm mass of size 3 x 2.5 cm, fungating, over the clitoral area. The inguinal nodes were palpable.

**Timeline of the current episode:** small swelling in the perineal region for 2 years, progressively increased to the present size.

**Figure 2:**
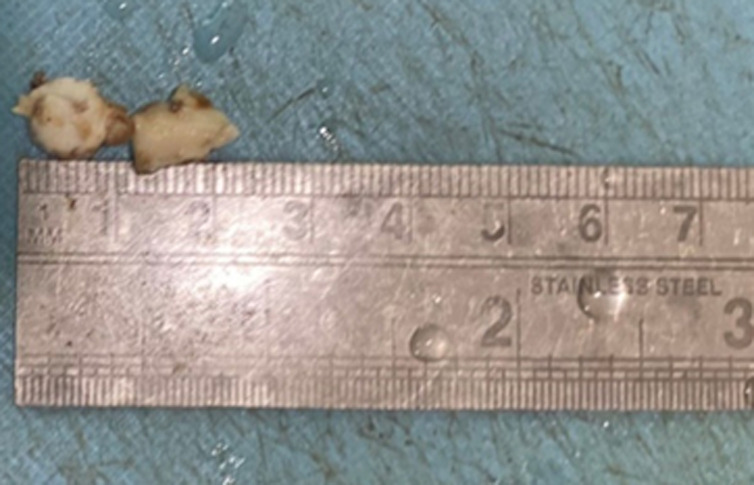
cut a section of biopsy from the clitoris

**Diagnostic assessment:** the patient was investigated further. The HCV and HBsAg test was nonreactive and HIV was negative. After obtaining written consent, the patient was shifted to operating theater for vulval biopsy and the specimen was sent to the histopathology section of the pathology department. The specimen received consisted of single containers labeled as a biopsy from the clitoris with labia majora. The container consists of two, irregular, whitish tissue pieces measuring approximately 0.5 x 0.5 cm each (Figure 2).

**Figure 3:**
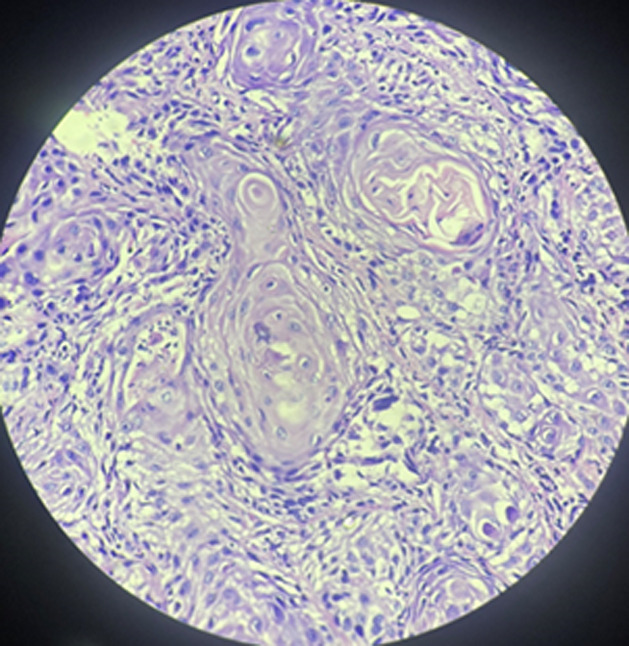
severe dysplastic squamous epithelial cells with eosinophilic cytoplasm and abundant keratin pearls

**Diagnosis:** a histological analysis showed the presence of severe dysplastic squamous epithelial cells with eosinophilic cytoplasm and abundant keratin pearls, proving the case of well-differentiated squamous cell carcinoma of the clitoris (Figure 3).

**Therapeutic interventions:** medications given to the patient during hospitalization are shown in Table 1.

**Table 1:** medications given to the patient during hospitalization

#	Medicine	Doses	Route	Days
1	Injection C Tax 1 GM	BD	IV	3 days
2	Injection Metro 100 CC	TDS	IV	3 days
3	Injection Pan 40 mg	BD	IV	3 days
4	Injection Tramadol	BD	IV	3 days

BD: *bis in die* (twice daily); TDS: *ter die sumendum* (three times a day); IV: intravenous line

**Follow-up and outcome of interventions:** complete surgical excision of the swelling and final histology documented the features suggestive of well-differentiated squamous cell carcinoma of clitoris.

**Patient perspective:** the patient was initially embarrassed due to the site of swelling but was finally satisfied with the early diagnosis and treatment.

**Informed consent:** the patient gave written informed consent so that this case report and any related photos could be published.

## Discussion

Clitoris cancer is extremely uncommon but significant due to its aggressive nature and early fatality during the clinical course. Because of the advanced stage upon presentation, managing such a case presents a difficulty for the surgical oncologist. Therefore, to prevent delays in identification and subsequent case treatment, such lesions necessitate an early biopsy [1]. Surgery as the primary treatment is among the therapy sequences linked to the best overall survival. Age, pathological grade, diagnosis stage, treatment order, and chemotherapy use were found to be independent predictors of outcome. The course of treatment with the highest overall survival rate is surgery alone. Every eligible patient should have access to surgery [4]. A rare subtype of vulvar squamous cell carcinoma with a favorable prognosis and a low incidence of groin metastases is called superficially invasive vulvar squamous cell carcinoma, or SISCCA. Regardless of the surgical method utilized, the pathology present, or the state of the surgical margins, SISCCA typically has a good prognosis [6].

## Conclusion

The diagnosis of a well-differentiated squamous cell carcinoma of clitoris is highlighted in this case study. The tumor was detected by examination and histological analysis, and surgical resection was the right course of treatment. An en-bloc resection of the inguinal lymph nodes was performed after a broad local excision. The surgeon faces difficulties in managing these cases because of the delayed diagnosis and absence of a widely recognized technique for handling them. After surgery, close observation showed no recurrence, highlighting the significance of routine follow-up in the management of such cases. By highlighting the significance of a precise diagnosis and multidisciplinary therapy for the best possible patient outcomes, this article contributes to the body of literature on well-differentiated squamous cell carcinoma of the cervix.
